# Higher premature atrial complex burden from the Holter examination predicts poor cardiovascular outcome

**DOI:** 10.1038/s41598-021-91800-4

**Published:** 2021-06-09

**Authors:** Ting-Chun Huang, Po-Tseng Lee, Mu-Shiang Huang, Pei-Fang Su, Ping-Yen Liu

**Affiliations:** 1 Institute of Clinical Medicine, College of Medicine, National Cheng Kung University, 1 University Road, Tainan City, Taiwan; 2 Division of Cardiology, Department of Internal Medicine, National Cheng Kung University Hospital, College of Medicine, National Cheng Kung University, 138 Sheng-Li Rd., North District, Tainan, 70403 Taiwan; 3 Department of Statistics, College of Management, National Cheng Kung University, Tainan, Taiwan

**Keywords:** Cardiovascular biology, Cardiovascular diseases, Arrhythmias

## Abstract

Premature atrial complexes (PACs) have been suggested to increase the risk of adverse events. The distribution of PAC burden and its dose–response effects on all-cause mortality and cardiovascular death had not been elucidated clearly. We analyzed 15,893 patients in a medical referral center from July 1st, 2011, to December 31st, 2018. Multivariate regression driven by ln PAC (beats per 24 h plus 1) or quartiles of PAC burden were examined. Older group had higher PAC burden than younger group (*p* for trend < 0.001), and both genders shared similar PACs distribution. In Cox model, ln PAC remained an independent risk factor for all-cause mortality (hazard ratio (HR) = 1.09 per ln PAC increase, 95% CI = 1.06‒1.12, *p* < 0.001). PACs were a significant risk factor in cause-specific model (HR = 1.13, 95% CI = 1.05‒1.22, *p* = 0.001) or sub-distribution model (HR = 1.12, 95% CI = 1.04‒1.21, *p* = 0.004). In ordinal PAC model, 4th quartile group had significantly higher risk of all-cause mortality than those in 1st quartile group (HR = 1.47, 95% CI = 1.13‒1.94, *p* = 0.005), but no difference in cardiovascular death were found in competing risk analysis. In subgroup analysis, the risk of high PAC burden was consistently higher than in low-burden group across pre-specified subgroups. In conclusion, PAC burden has a dose response effect on all-cause mortality and cardiovascular death.

## Introduction

Premature atrial complexes (PACs) are related to early depolarization of the atrial myocardium, and they are a very common arrhythmic disturbance in the general population^1^. PACs originating form pulmonary veins^2,3^ or other thoracic veins^4^ are highly associated with spontaneous initiation of atrial fibrillation (AF), and nowadays, catheter ablation in these origins prevents recurrence of AF. Symptoms of patients with PACs range from fatigue, dyspnea, and dizziness, to near fainting, while some are asymptomatic. The long-term prognosis of these patients is characterized by increased risk of stroke, cardiovascular morbidities, and mortality due to the burden of PAC itself or to subsequent AF^5–8^. In the latest meta-analysis ^9^, frequent PACs were associated with AF [hazard ratio (HR) 2.96, 95% confidence interval (CI) 2.33‒3.76], first stroke (HR 2.54, 95% CI 1.68‒3.83), and all-cause mortality (HR 2.14, 95% CI 1.94‒2.37).

Currently, the distribution of PAC burden in different age group and gender was not clearly exhibited. Although PACs were reported to be a risk factor for all-cause mortality^9^, the dose–response effects on all-cause mortality and cardiovascular death had not been fully elucidated. The competing risk of cardiovascular death and non-cardiovascular death is another important and emerging aspect of survival analysis^10^. In this study, we enrolled a hospital-based East Asian cohort of more than 20,000 patients who had undergone 24-h Holter monitoring to exhibit the distribution of PACs, and collected all relevant clinical information, to investigate the effects of PAC burden on all-cause mortality by different models and cardiovascular death by competing risk model.

## Results

### Baseline characteristics of the study cohort

**Figure 1:**
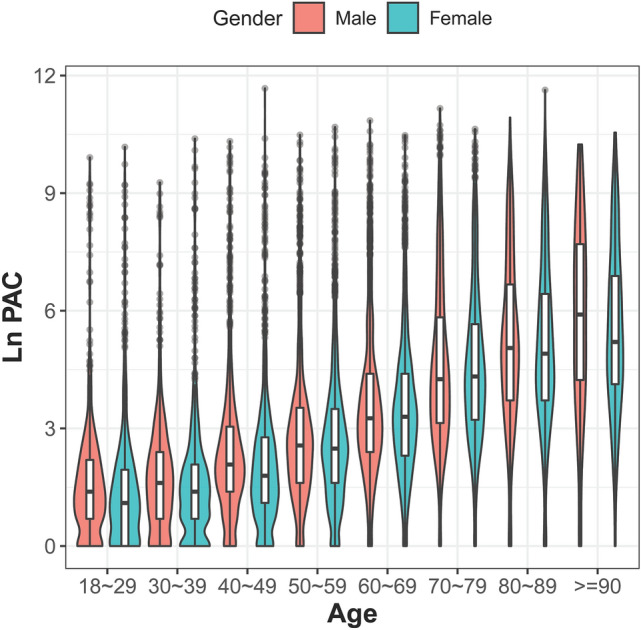
Violin plot. The PAC distribution is presented by age and gender. The PAC burden increased with age (*p* for trend < 0.05), and similar distribution across all age bracket was found in both gender. The white bar in each violin plot presents interquartile range, and the central black horizontal line is median. The black lines stretched from the bar are the lower and upper adjacent values defined as first quartile − 1.5 IQR and third quartile + 1.5 interquartile range respectively. The colored area is the density plot, and the width suggests the frequency.

The distribution of PAC burden was presented in the violin plot (Fig. 1), by age and gender. No obvious gender difference was found in each age bracket. Bi-modal distribution of PAC burdens in each group were noted. These peaks were closer in younger age and more apart from each other in older age. Simultaneously, higher median and wider interquartile range were found in older age bracket (*p* for trend < 0.001 for both gender groups), and both genders shared similar feature across each age group (supplementary table S1).

Baseline characteristics are presented in Table 1, overall and by event. The study cohort was composed of 15,893 patients, with a median follow-up period of 924.1 days, and 905 patients died of any cause (event group). Compared to the event-free group, patients in the event group were older (71.5 ± 13.0 vs. 56.5 ± 17.4 years, *p* < 0.001, Table 1) and had higher proportion of males (58.9% vs. 44.2%, *p* < 0.001) and higher burden of PACs. Moreover, event group patients had much more comorbidities, including hypertension, diabetes mellitus (DM), heart failure (HF), acute myocardial infarction, coronary artery disease (CAD), peripheral arterial disease, stroke, chronic kidney disease (CKD), and hypertrophic cardiomyopathy. Regarding medications, aspirin, P_2_Y_12_ inhibitor, angiotensin-converting enzyme inhibitors (ACEi), angiotensin receptor blockers (ARB), dihydropyridine calcium channel blocker, non-dihydropyridine calcium channel blocker, diuretics, anticoagulants, and class III antiarrhythmic drugs were prescribed more in the event group. The proportion of dyslipidemia and the use of beta blocker, class I antiarrhythmic medication were not different between these two groups.

**Table 1:** Demographic and clinical characteristics of overall study cohort, and event and event-free groups.

	Event (N = 905)	Event-free (N = 14,988)	*p*
Age, y, mean (SD)	71.5 (13.0)	56.5 (17.4)	< 0.01
Male, N (%)	533 (58.9)	6625 (44.2)	< 0.01
Follow-up days*	862.8 (431.6)	927.8 (442.5)	< 0.01
PAC, mean (SD)	1356.1 (5137.2)	522.6 (2824.3)	< 0.01
HTN, N (%)	609 (67.3)	6582 (43.9)	< 0.01
DM, N (%)	361 (39.9)	2892 (19.3)	< 0.01
Dyslipidemia, N (%)	426 (47.1)	6774 (45.2)	0.27
HF, N (%)	232 (25.6)	1140 (7.6)	< 0.01
AMI, N(%)	65 (7.2)	456 (3.0)	< 0.01
CAD, N (%)	187 (20.7)	1457 (9.7)	< 0.01
PAOD, N (%)	43 (4.8)	254 (1.7)	< 0.01
Stroke, N (%)	141 (15.6)	1030 (6.9)	< 0.01
CKD, N (%)	431 (47.6)	2181 (14.6)	< 0.01
HCM, N (%)	27 (3.0)	242 (1.6)	< 0.01
Aspirin, N (%)	231 (25.5)	2910 (19.4)	< 0.01
P_2_Y_12_ inhibitor, N (%)	113 (12.5)	1081 (7.2)	< 0.01
Warfarin, N (%)	20 (2.2)	124 (0.8)	< 0.01
NOAC, N( %)	8 (0.9)	57 (0.4)	< 0.05
ACEi/ARB, N (%)	184 (20.3)	2172 (14.5)	< 0.01
DHP CCB, N (%)	253 (28.0)	2369 (15.8)	< 0.01
Diuretics, N (%)	213 (23.5)	1058 (7.1)	< 0.01
Class I AAD, N (%)	5 (0.6)	103 (0.7)	0.83
Class III AAD, N (%)	62 (6.9)	316 (2.1)	< 0.01
Beta blocker, N (%)	169 (18.7)	3039 (20.3)	0.25
Non-DHP CCB, N (%)	59 (6.5)	664 (4.4)	< 0.01

Abbreviations: *AAD* antiarrhythmic drug, *ACEi/ARB* angiotensin-converting enzyme inhibitor/angiotensin receptor blocker, *AMI* acute myocardial infarction, *CAD* coronary artery disease, *CKD* chronic kidney disease, *DHP CCB* dihydropyridine calcium channel blocker, *DM* diabetes mellitus, *HCM* hypertrophic cardiomyopathy, *HF* heart failure, *HTN* hypertension, *Non-DHP CCB* non-dihydropyridine calcium channel blocker, *PAOD* peripheral arterial occlusive disease, *PAC* premature atrial complex, *PVC* premature ventricular complex, *NOAC* novel oral anticoagulant.

*Represents median follow-up days(SD).

Baseline characteristics of each quartile were presented in Table 2. Briefly speaking, groups of higher PAC burden were significantly older, and had higher proportion of male, more comorbidities, and more prescriptions.

**Table 2:** Demographic and clinical characteristics of each quartile group.

	1st Quartile (N = 3735)	2nd Quartile (N = 4169)	3rd Quartile (N = 4004)	4th Quartile (N = 3985)	*p*
Age, y, mean (SD)	43.5 (15.5)	52.8 (15.0)	62.8 (13.7)	69.6 (14.1)	0.01
Male, N (%)	1468 (39.3)	1936 (46.4)	1865 (46.6)	1889 (47.4)	0.01
Follow-up days*	916.0 (443.2)	948.1 (438.9)	932.3 (439.3)	898.4 (446.0)	0.01
PAC, mean (SD)	1.3 (1.1)	8.4 (3.4)	35.3 (16.2)	2228.1 (5698.5)	0.01
HTN, N (%)	990 (26.5)	1630 (39.1)	2108 (52.6)	2463 (61.8)	0.01
DM, N (%)	462 (12.4)	752 (18.0)	980 (24.5)	1059 (26.6)	0.01
Dyslipidemia, N (%)	1281 (34.3)	1886 (45.2)	2055 (51.3)	1978 (49.6)	0.01
HF, N (%)	179 (4.8)	281 (6.7)	372 (9.3)	540 (13.6)	0.01
AMI, N (%)	86 (2.3)	123 (3.0)	143 (3.6)	169 (4.2)	0.01
CAD, N (%)	212 (5.7)	343 (8.2)	497 (12.4)	592 (14.9)	0.01
PAOD, N (%)	32 (0.9)	50 (1.2)	86 (2.1)	129 (3.2)	0.01
Stroke, N (%)	127 (3.4)	240 (5.8)	345 (8.6)	459 (11.5)	0.01
CKD, N (%)	269 (7.2)	496 (11.9)	727 (18.2)	1120 (28.1)	0.01
HCM, N (%)	29 (0.8)	63 (1.5)	68 (1.7)	109 (2.7)	0.01
Aspirin, N (%)	412 (11.0)	736 (17.7)	960 (24.0)	1033 (25.9)	0.01
P_2_Y_12_ inhibitor, N (%)	149 (4.0)	256 (6.1)	351 (8.8)	438 (11.0)	0.01
Warfarin, N (%)	14 (0.4)	32 (0.8)	44 (1.1)	54 (1.4)	0.01
NOAC, N (%)	4 (0.1)	11 (0.3)	12 (0.3)	38 (1.0)	0.01
ACEi/ARB, N (%)	340 (9.1)	519 (12.4)	691 (17.3)	806 (20.2)	0.01
DHP CCB, N (%)	305 (8.2)	550 (13.2)	750 (18.7)	1017 (25.5)	0.01
Diuretics, N (%)	145 (3.9)	238 (5.7)	350 (8.7)	538 (13.5)	0.01
Class I AAD, N (%)	12 (0.3)	16 (0.4)	19 (0.5)	61 (1.5)	0.01
Class III AAD, N (%)	54 (1.4)	82 (2.0)	91 (2.3)	151 (3.8)	0.01
Beta blocker, N (%)	605 (16.2)	846 (20.3)	893 (22.3)	864 (21.7)	0.01
Non-DHP CCB, N (%)	112 (3.0)	158 (3.8)	192 (4.8)	261 (6.5)	0.01

Abbreviations: *AAD* antiarrhythmic drug, *ACEi/ARB* angiotensin-converting enzyme inhibitor/angiotensin receptor blocker, *AMI* acute myocardial infarction, *CAD* coronary artery disease, *CKD* chronic kidney disease, *DHP CCB* dihydropyridine calcium channel blocker, *DM* diabetes mellitus, *HCM* hypertrophic cardiomyopathy, *HF* heart failure, *HTN* hypertension, *Non-DHP CCB* non-dihydropyridine calcium channel blocker, *PAOD* peripheral arterial occlusive disease, *PAC* premature atrial complex, *NOAC* novel oral anticoagulant.

*Represents median follow-up days(SD).

### Risk factors for all-cause mortality

By using univariate analysis, we found that ln PAC could predict all-cause mortality (odds ratio (OR) = 1.27, 95% CI = 1.24‒1.31, *p* < 0.001). After adjustment for age, gender, comorbidities (DM, hypertension, stroke, CAD, CKD, HF), and medications (aspirin), the multivariate analysis model revealed that ln PAC remained a significant risk factor for all-cause mortality (Table 3, OR = 1.07 per ln PAC increase, 95% CI = 1.03‒1.10, *p* < 0.001).

**Table 3:** Multivariate survival analyses of all-cause mortality and competing risk of cardiovascular death.

	Cox proportional model*	Cause-specific model*	Subdistribution model*
Ln PAC	1.09 (1.06‒1.12, *p* < 0.001)	1.13 (1.05‒1.22, *p* = 0.001)	1.12 (1.04‒1.21, *p* = 0.004)
PAC < 4	**–**	–	**–**
4 ≤ PAC < 16	1.03 (0.78‒1.36, *p* = 0.830)	0.90 (0.43‒1.90, *p* = 0.787)	0.89 (0.42‒1.87, *p* = 0.760)
16 ≤ PAC < 77	1.25 (0.96‒1.62, *p* = 0.094)	1.36 (0.70‒2.65, *p* = 0.364)	1.37 (0.69‒2.74, *p* = 0.360)
PAC ≥ 77	1.67 (1.29‒2.15, *p* < 0.001)	1.88 (0.99‒3.59, *p* = 0.055)	1.82 (0.92‒3.62, *p* = 0.088)

*Adjusted for age, gender, DM, hypertension, CAD, CKD, HF, stroke, aspirin, and ACEi/ARB.

Abbreviations: *ACEi/ARB* angiotensin-converting enzyme inhibitor/angiotensin receptor blocker, *CAD* coronary artery disease, *CKD* chronic kidney disease, *DM* diabetes mellitus, *HF* heart failure, *HR* hazard ratio, *PAC* premature atrial complex.

Ordinal PAC model stratified by quartiles of PAC burden was adjusted for the same factors as in ln PAC model (Table 3). The adjusted risk of all-cause mortality significantly increased more in 4th quartile than 1st quartile (OR = 1.47, 95% CI = 1.13‒1.94, *p* = 0.005).

### Survival analysis

Though the current analysis showed that higher PAC burden was a predictor of all-cause mortality, to ensure the reliability of our findings, we performed multivariate Cox proportional-hazard model analysis. Herein, we found that ln PAC was still independently associated with all-cause mortality (Table 3, HR = 1.09 per ln PAC increase, 95% CI = 1.06‒1.12, *p* < 0.001) after adjustment for the same factors mentioned above plus use of ACEi or ARB.

**Figure 2:**
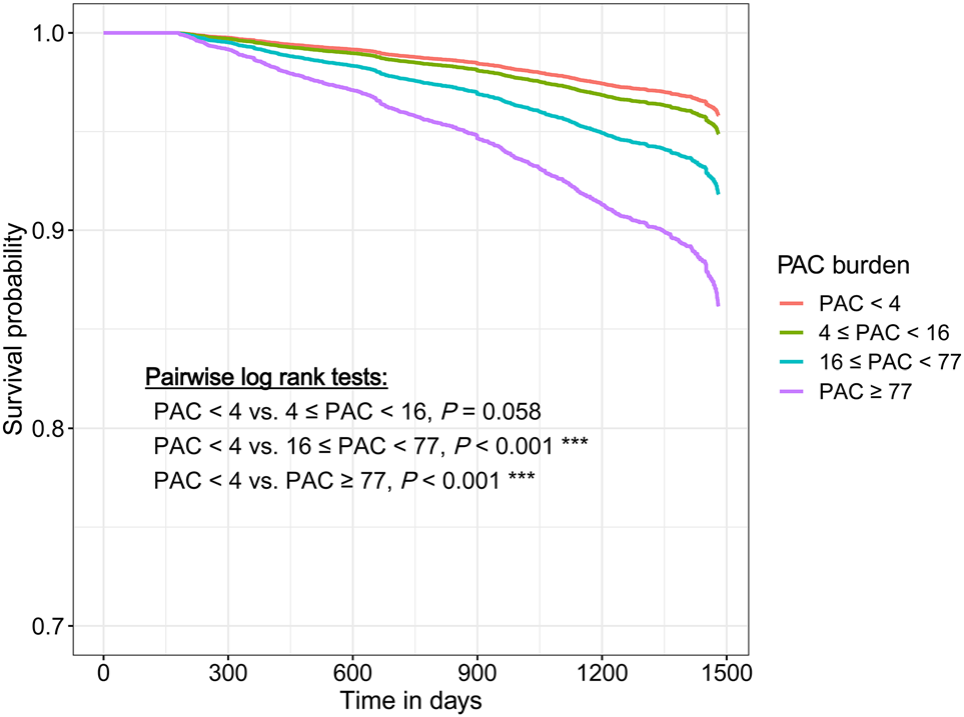
Survival curve of the study subjects. Compared to 1st quartile, higher PAC burden groups (quartile 3rd and 4th) had significantly lower survival probability. Abbreviation: PAC, premature atrial complex.

After multivariate adjustment, patients in 4th quartile group had significantly higher risk of all-cause mortality than those in 1st quartile group (Table 3, HR = 1.67, 95% CI = 1.29‒2.15, *p* < 0.001). In Fig. 2, the survival probability of each high PAC burden group (3rd and 4th quartile) was significantly lower than that of reference group (1st quartile) during the entire follow-up period.

### Competing risk of mortality

PAC burden itself has been proven to be a risk factor for all-cause mortality. PACs are common cardiac dysrhythmic disturbances, and we speculated on whether PAC burden can lead to more cardiovascular death. We thus performed multivariate cause-specific model analysis to evaluate the effect of PAC on the rate of occurrence of cardiovascular death in subjects who were event-free at the time of study, and a sub-distribution model analysis to estimate the effect of PAC on the absolute risk of cardiovascular death during the entire follow-up period.

The models, which were driven by ln PAC, interestingly, showed that PACs increase the risk of cardiovascular death in surviving patients (Table 3, HR = 1.13 per ln PAC increase, 95% CI = 1.05‒1.22, *p* = 0.001) or the overall incidence of cardiovascular death (HR = 1.12 per ln PAC increase, 95% CI = 1.04‒1.21, *p* = 0.004). While considering ordinal PAC model, high PAC burden groups shared the same risk of cardiovascular death compared to 1st quartile group (Table 3).

### Subgroup analysis

**Figure 3:**
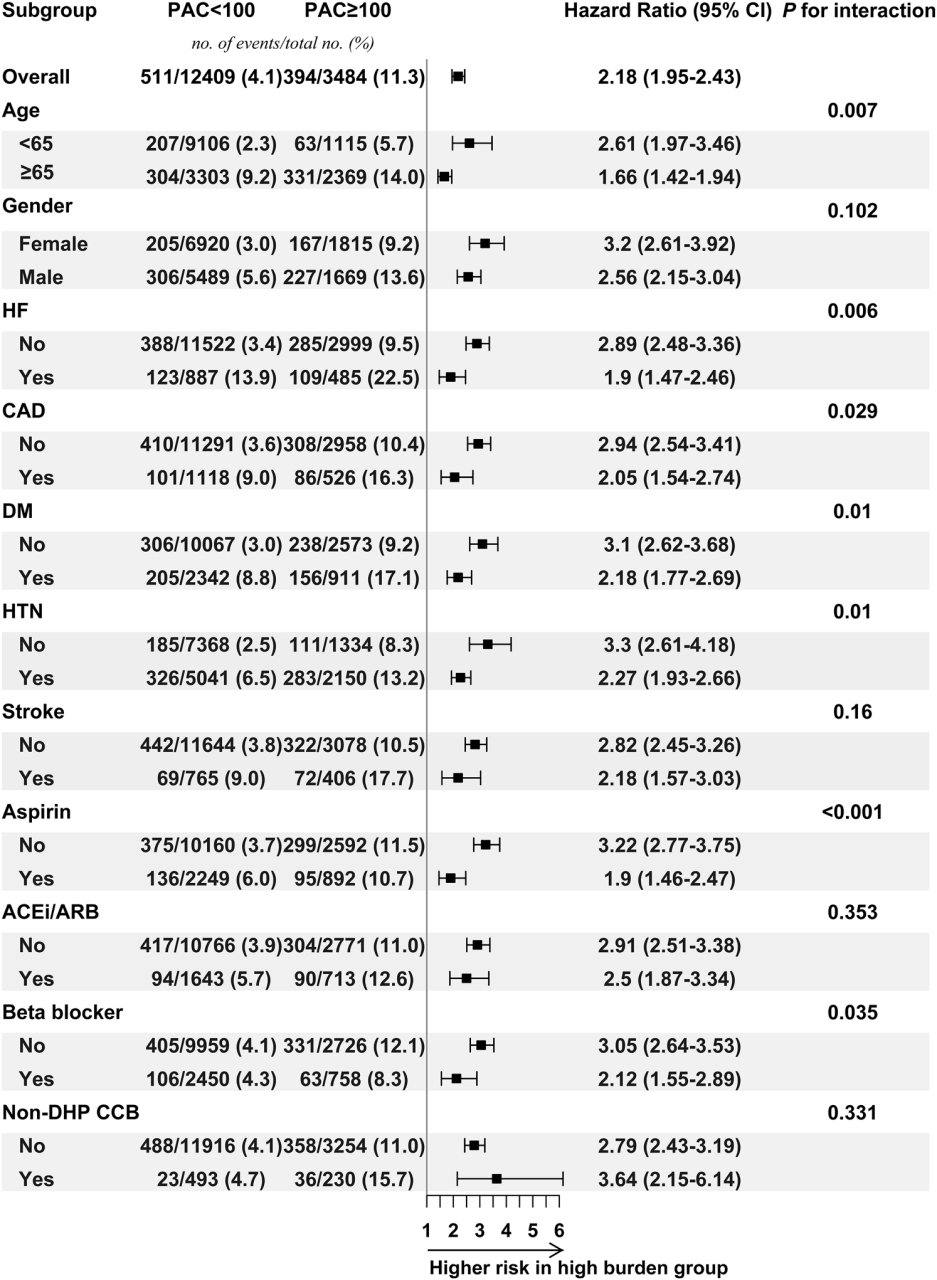
Subgroup analysis. Patients were divided into two groups based on a PAC cut-off value of 100. High-burden group had greater risk of all-cause mortality than low-burden group, and this finding was consistent across all pre-specified groups. Abbreviation: ACEi/ARB, angiotensin-converting enzyme inhibitor/angiotensin receptor blocker; CAD, coronary artery disease; CI, confidence interval; DM, diabetes mellitus; HF, heart failure; Non-DHP CCB, non-dihydropyridine calcium channel blocker; PAC, premature atrial complex.

We subsequently divided our cohort into two groups, i.e. high burden group (PAC ≥ 100 beats per 24 h) and low burden group (PAC < 100 beats per 24 h), to investigate the relative risk in different subgroups (Fig. 3). Because of significant higher risk in high burden group and convenience for clinical use, we choose 100 (78th percentile) as a specific cut-off value. The risks of high PAC burden were consistently higher than in the low burden group across the overall cohort and pre-specified subgroups. In each pre-specified group, the incidence of mortality was still highest in high PAC burden patients combined with older age or comorbidity, e.g. HF, CAD, DM, or hypertension than high PAC burden patients who were younger or comorbidity-free. However, high PAC burden patients younger than 65 years-of-age or in the subgroups free of pre-specified comorbidities had higher relative risk than those older than 65 years-of-old or with comorbidities. Notably, lower relative risk was found in high burden group patients who were using aspirin, and beta blockers than those patients without use of aspirin or beta blockers.

## Discussion

In this study, we exhibited clearly an increasing trend in PAC burdens with age and no obvious gender difference in this largest hospital-based 24-h Holter monitoring cohort. We investigate the risk of all-cause mortality in patients with PACs. Comorbidities and prescriptions were comprehensively evaluated. Our study demonstrated that PAC did increase the risk of all-cause mortality and decrease the survival probability during follow-up, no matter which models (driven by ln PAC or ordinal PAC Cox proportional model) were analyzed. Furthermore, PACs did increase the risk rate of cardiovascular death during the entire follow-up period. In subgroup analyses, the risk of high PAC burden was consistent across overall cohort and pre-specified subgroups.

Conen et al^1^ had studied a Swiss cohort and found that the frequency of PACs steadily increased with age, with rates of 0.8, 1.1, 1.4, 2.3, and 2.6 PACs per h among participants aged 50–55, 55–60, 60–65, 65–70, and ≥ 70 years, respectively. Our study also demonstrated age dependent feature of PACs. In literature, several studies mainly focused on AF, and aging related oxidative stress, calcium dysregulation, atrial myocyte apoptosis, and atrial fibrosis all contribute AF initiation and/or maintenance^11^. However, the detailed mechanism of aging promotes atrial remodeling or degeneration, the initiation of PAC, the transition from PAC to AF are still not fully elucidated.

PAC is a quite common arrhythmia among both outpatients and inpatients. Although several studies have reported that PACs increase the risk of all-cause mortality, stroke, and AF, we found that most previous studies used dichotomous method to calculate the hazard relationships^6,7,10,12–14^. In those studies, because PAC burden was right-skewed distributed and not normally distributed, the specific cut-off values were usually less than 100 beats per 24 h. Our ordinal model exhibited that PAC more than 77 beats per 24 h (4th quartile) had more risk of all-cause mortality than 1st quartile group, but all ordinal PAC groups shared the same risk of cardiovascular death. The possible reason is that all of the confidence intervals for the different PAC groups overlap in the competing risk analysis driven by ordinal PAC groups.

Models driven by ln PAC clearly presented the dose–response effect of PAC burden on all-cause mortality and cardiovascular death in our study. Dewland et al^15^ used a community-based cohort of 1260 patients to evaluate long-term prognosis by analyzing PACs by ordinal groups or beats per hour. We demonstrated that patients with higher PAC burdens truly had more risks by using a hospital-based cohort of nearly 16,000 patients, and such results are more in line with our daily practice.

PACs, as precursors arising in the thoracic veins^2–4^, are important for initiation and perpetuation of AF, and the main focal mechanisms of PAC, including enhanced automaticity, early afterdepolarization, or delayed afterdepolarization, are highly related to adrenergic activation^16^, which is usually provoked by chronic inflammation^17^. Chronic inflammation is closely associated with AF^18^, which is well-known as a risk factor for cardiovascular death. PACs did be competing risks of cardiovascular death in our cohort, and PACs itself could be a sensitive maker of systemic inflammation and a warning sign in the very early phase of atrial cardiomyopathy. Anti-inflammation is one of the primary effects of aspirin and beta blockers. Indeed, all these medications potentially played pharmaceutical protective roles in our cohort.

In our subgroup analysis, we found that high PAC burden was a general risk factor for all-cause mortality across all pre-specified subgroups. The incidence of events (all-cause mortality) was still highest in patients with comorbidity and higher PAC burdens. Interestingly, high PAC burden had higher relative risk than low PAC burden in younger and comorbidity-free group, whereas, younger and comorbidity-free patients are usually considered to have lower risk of adverse events. The possible explanation is that, firstly, low incidence of events in comorbidity-free group with low PAC burden. Secondly, high PAC burden as an indicator of sympatho-vagal imbalance and/or a phenotype of chronic atrial inflammation may contribute to similar health risk as other well-recognized cardiovascular disease.

Several specific underlying diseases or medications, such as chronic lung disease itself and use of bronchodilators, or use of anxiolytics, could be associated with altered burdens of PAC which might lead to another specific cut-off for this subgroup. Dr. Kusunoki et al. studied atrial and ventricular arrhythmia in stable chronic obstructive lung disease and used > 100 beats per 24 h as a specific cut-off. They concluded that increased supraventricular premature complexes, or PACs, might be strongly associated with the use of bronchodilator^19^. However, another group studied the combination of theophylline and salbutamol, and also concluded that oral theophylline added to a regimen of salbutamol does not seem to affect the occurrence or severity of arrhythmias^20^. In our current study, we had focused on the whole Holter cohort, tried to collect all relevant information, including history of cardiovascular comorbidities and medications which had be proved to improve long-term prognosis, and evaluated the impact of PACs on prognosis.

A number of cohorts have been observed that higher PAC burdens had higher probability of developing AF and stroke^9^. The mortality rate of patients with AF is higher than those without AF. In literature, no studies have ever addressed this issue about the disease status transition, e.g. causal mediation analysis, in patients with PACs. It deserved more attention to investigate the sophisticated interaction among AF, stroke, and mortality in patients with PACs.

How to treat patients with high PAC burdens is an unmet clinical need. It is worth mentioning that beta blockers prescribed for relieving symptoms potentially had protective effects. Owing that the mortality could be reduced in patients taking beta blockers, this encouraged our finding in consistent across from the low over the high PAC burden groups.

### Limitations

First, this study was a single-hospital-based retrospective study in which we might have lost some information on primary endpoints. However, we only enrolled patients who had been followed up for more than 180 days, and the median of overall cohort follow-up duration was 924.1 days. All information obtained helped us to construct a reliable and consistent model of survival analysis. Second, lots of confounders had interference in the main outcome, i.e. all-cause mortality. We used different models and the results were quite consistent. Third, in our current study, we did not completely analyze the whole possible medications associated with PAC events, but only focused on the Holter cohort and outcome associated medical history alone. This might have bias in studying the effect of chronic lung disease and chest medications. Fourth, our findings were unable to answer a question about the disease trajectory state transition from higher PAC burden to AF, stroke, and cardiovascular death. Further research is needed.

## Methods

### Databank

We conducted a retrospective cohort study by using Cardiovascular Disease Databank from National Cheng-Kung University Hospital^19–21^ to enroll consecutive patients who had ever undergone 24-h Holter monitoring. Our databank contained the complete electronic medical records of patients who had been admitted to our cardiovascular ward/coronary care unit and had been followed up at cardiovascular outpatient department, or had undergone cardiovascular studies, including echocardiography, 24-h Holter monitoring, treadmill exercise test, thallium scan, peripheral or coronary angiography with/without intervention, electrocardiography with/without ablation, or device therapy. All patients’ longitudinal data on demographics, symptoms, laboratory data, medications, and imaging studies from January 1st, 2009, till December 31st, 2018, were all collected. This study complied with the Declaration of Helsinki and was approved in National Cheng Kung University Hospital by an independent ethics committee (A-ER-107-149, A-ER-108-381), and the informed consent was waived because of the retrospective nature of this study. The Cardiovascular Disease Databank was built up based on the collected data from the study of Artificial Intelligence with Deep Learning and Genes on Cardiovascular Disease, with ClinicalTrials.gov Identifier NCT03877614.

### Databank validation

We also conducted a pilot validation study which randomly enrolled 200 patients, and 4 random traits, such as baseline characteristics, comorbidities, or medications, per patient, were manually reviewed by two cardiovascular physicians. The accuracy rate of all traits was 99.12% (793/800).

### Study cohort

**Figure 4:**
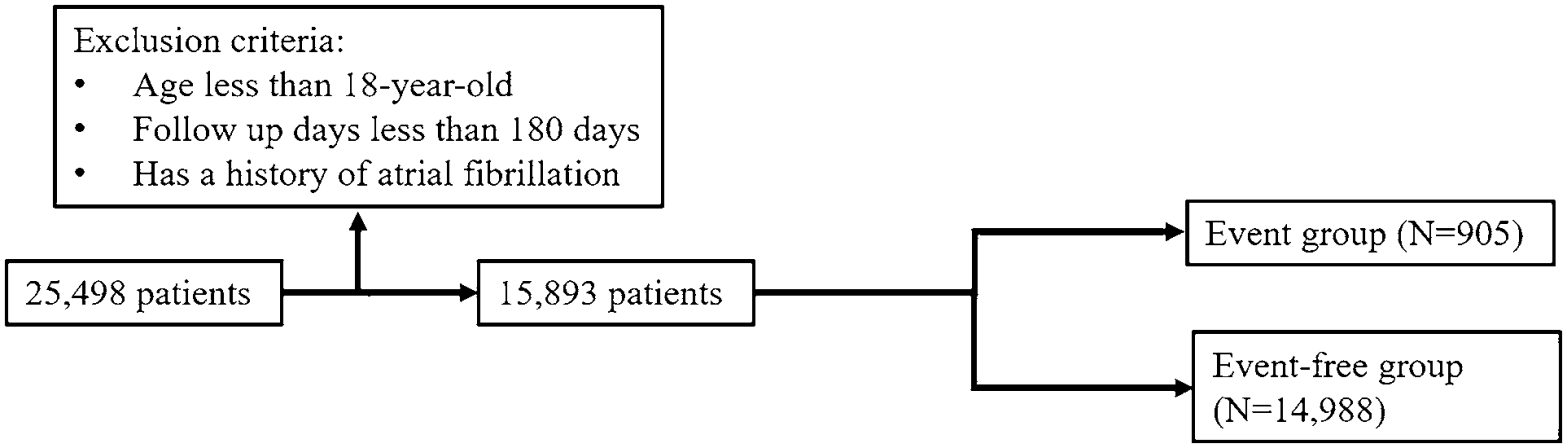
Study cohort. A cohort study enrolled 15,893 consecutive patients after excluding patients aged less than 18 years, followed up for less than 180 days, or having a history of atrial fibrillation. The total number of all-cause mortality was 905 during the entire follow-up period, and these patients were denoted event group.

We consecutively analyzed the hospital electronic medical record quantitative database in a single medical referral center from July 1st, 2011, to December 31st, 2018, with 30,488 records of Holter monitoring from 25,398 patients. Patients aged less than 18 years and who were followed up for less than 180 days were excluded in the study. 24,071 Holter monitoring records belonging to 19,528 patients were analyzed. If patients had repeated examinations, we used the earliest PAC burdens and clinical information for analysis. We excluded 3635 patients who had a history of atrial fibrillation documented by electrocardiography or 24-h Holter recording before the indexed Holter examination. Finally, 15,893 patients were included in this study cohort (Fig. 4).

### Definition of clinical characteristics and endpoints

The primary endpoint of this study was all-cause mortality. All patients were divided into event group (which comprised patients who died of any cause) and event-free group. Baseline characteristics, comorbidities, and medications were all recorded on the date of enrollment. To ensure the accuracy of patients’ diagnoses, each variable was determined comprehensively based on the doctor's manual input, laboratory results, corresponding treatment, and International Classification of Diseases (ICD) codes (Supplementary Table S2). All used mediations were defined, and prescription duration was more than 16 weeks (from 4 weeks before index Holter monitoring to 16 weeks after index Holter monitoring). Mortality data were retrieved from the Collaboration Center of Health Information Application (CCHIA), Ministry of Health and Welfare in Taiwan, and further confirmed by linking with the National Death Registry. Cause of death was reviewed by one cardiologist (P. T. Lee) and validated by the other two cardiologists (T. C. Huang and M. S. Huang). Classification of deaths as cardiovascular or non-cardiovascular was aimed at capturing the primary cause of death. The primary cause of death was defined as the underlying disease or injury that initiated the course of events that resulted in death. Cardiovascular death was defined as death due to an event of acute myocardial infarction, sudden cardiac death, heart failure, stroke, cardiovascular procedures, cardiovascular hemorrhage, or other cardiovascular causes^20–22^.

### 24-h Holter monitoring

All patients were asked to follow their daily routines without any limitation during the recording period. We used DR200/HE Holter^21–23^ (NorthEast Monitoring, Inc., Maynard, MA USA) with a frequency response of 0.05 to 70 Hz in 180 samples/sec mode. We used 7-lead placement to acquire three-channel information: V5 (−, right manubrium; +, left anterior axillary line on the 5th rib), V1 (−, left of the manubrium; +, 2 cm right of the xiphoid process), and lead III (−, centered on the manubrium; +, left of the mid-clavicular line on the 5th rib).

All recordings were analyzed by using Holter LX Analysis (NorthEast Monitoring, Inc., Maynard, MA USA). This system was programmed to automatically capture all ectopic beats or rhythmic disturbances, and the recordings were reviewed by experienced technicians. PAC and PVC (premature ventricular complex) were defined as coupling interval < 90% and < 80% of the last coupling interval, respectively; supraventricular and ventricular tachycardia episodes were defined as three or more consecutive supraventricular or ventricular beats, respectively, at a speed of more than 120 beats per minute. A PAC or a supraventricular event was considered when QRS duration was less than 120 ms unless aberrant morphology of QRS, otherwise would be thought of as a PVC or a ventricular tachycardia event. The total number of PACs was summed during the monitoring period (beats per 24 h). All the arrhythmic episodes, unknown strips, and final formal 24-h Holter reports were reviewed and confirmed by qualified senior cardiologists.

### Statistics analysis

Categorical variables were presented as frequencies and percentages, whereas continuous variables were reported as means and standard deviations. Because of the right-skewed distribution of PAC (beats per 24 h), we used nature-log transformation of PAC plus 1 for further analysis (denoted as ln PAC). We presented the distribution of PAC burden by age bracket and gender, and used Jonckheere–Terpstra test to examine whether PAC burden is dependent of age.

Besides of ln PAC, we divided patients into quartiles (PAC < 4, 4 ≤ PAC < 16, 16 ≤ PAC < 77, and PAC ≥ 77 beats per 24 h) for analysis. To clarify the association between all-cause mortality and corresponding risk factors, categorical variables were compared using χ^2^ test, and continuous variables were compared with Mann–Whitney test preliminarily. Then, risk factors were analyzed with univariate and multivariate logistic regression. Regression coefficient and odds ratio (OR) were calculated for each independent risk factor.

We then performed the analysis of survival data. The primary endpoint (that is, all-cause mortality) was analyzed using the Cox-proportional hazard model, and all risk variables were selected by univariate analysis with *p* value < 0.05. HR with 95% CI was calculated for each independent risk factor. In addition, considering non-cardiovascular death as a competing risk, we used cause-specific hazard model to investigate the effect of PAC on the rate of occurrence of cardiovascular death in subjects who were event-free at the time of study, and sub-distribution model analysis to estimate the effect of PAC on the absolute risk of cardiovascular death during the entire follow-up period. Similarly, demographic and clinical variables with univariate *p* value < 0.05 were used for multivariate analyses.

All statistical tests were 2-sided, and *p* value less than 0.05 was considered statistically significant. All analyses were performed with statistical software R, version 3.6.3 for Windows.

## Conclusion

In this study, we found that PAC is a common but not absolutely benign arrhythmic disturbance. The risks of all-cause mortality and cardiovascular death increased with PAC burdens. 24-h Holter monitoring could provide extra hints of patients’ general conditions.

## Supplementary Information

Supplementary Information.

## Abbreviations

ACEi: Angiotensin converting enzyme inhibitor
AF: Atrial fibrillation
ARB: Angiotensin receptor blocker
CAD: Coronary artery disease
CI: Confidence interval
CKD: Chronic kidney disease
DM: Diabetes mellitus
HF: Heart failure
HR: Hazard ratio
PAC: Premature atrial complexes
