# Supplementary material for: Higher premature atrial complex burden from the Holter examination predicts poor cardiovascular outcome
Source: Sci Rep. 2021 Jun 9;11:12198. doi: 10.1038/s41598-021-91800-4 (PMC8190115; doi:10.1038/s41598-021-91800-4)
Supplement: Supplementary file 1 — Supplementary Information. [file 41598_2021_91800_MOESM1_ESM.pdf]

Higher premature atrial complex burden from the Holter examination predicts poor cardiovascular outcome

Brief title: PACs increase the risk of mortality

Ting-Chun Huang, M.D. [a,b], Po-Tseng Lee, M.D. [a,b], Mu-Shiang Huang, M.D. [b], Pei-Fang Su, Ph.D. [c], Ping-Yen Liu, M.D., Ph.D. [a,b,*]

[a] Institute of Clinical Medicine, College of Medicine, National Cheng Kung University, Tainan, Taiwan
[b] Division of Cardiology, Department of Internal Medicine, National Cheng Kung University Hospital, College of Medicine, National Cheng Kung University, Tainan, Taiwan
[c] Department of Statistics, College of Management, National Cheng Kung University, Tainan, Taiwan

Supplementary Table S1: Premature atrial complex burdens (presented as ln PAC) in each age bracket and gender

Age	Gender	n	Median	Mean	SD	Min	Max	IQR
18~29	Male	639	1.39	1.62	1.58	0.00	9.91	1.50
18~29	Female	822	1.10	1.46	1.63	0.00	10.17	1.95
30~39	Male	554	1.61	1.78	1.58	0.00	9.27	1.70
30~39	Female	836	1.39	1.64	1.66	0.00	10.39	1.39
40~49	Male	893	2.08	2.43	1.89	0.00	10.32	1.66
40~49	Female	1217	1.79	2.10	1.78	0.00	11.67	1.67
50~59	Male	1468	2.56	2.87	1.91	0.00	10.48	1.92
50~59	Female	1917	2.48	2.71	1.80	0.00	10.68	1.89
60~69	Male	1632	3.26	3.59	1.98	0.00	10.85	2.00
60~69	Female	1820	3.30	3.49	1.83	0.00	10.47	2.09
70~79	Male	1194	4.26	4.61	2.15	0.00	11.16	2.70
70~79	Female	1366	4.32	4.57	2.00	0.00	10.63	2.44
80~89	Male	701	5.05	5.19	2.22	0.00	10.93	2.96
80~89	Female	692	4.91	5.09	2.17	0.00	11.63	2.72
≥90	Male	77	5.90	5.75	2.52	0.00	10.24	3.46
≥90	Female	65	5.20	5.53	2.14	0.00	10.54	2.76

Abbreviations: Min: minimum, Max: maximum, SD: standard deviation

Supplementary Table S2: Definitions of terms

	Definitions
**Hypertension**	Documented in medical records of outpatient department or discharge note and/or concurrent use of calcium channel blockers
**Diabetes mellitus**	Documented in medical records of outpatient department or discharge note and/or HbA1c ≧7.0% and/or concurrent use of anti-diabetic drugs
**Hyperlipidemia**	Documented in medical records of outpatient department or discharge note and/or concurrent use of statin and/or abnormal lipid panels (LDL ≧160, TG ≧150, total cholesterol ≧200 mg/dL)
**Chronic kidney disease**	Documented in medical records of outpatient department or discharge note and/or eGFR ≦60 mL/min/1.73m^2
**Atrial fibrillation**	Documented atrial fibrillation in 12-lead surface electrocardiogram or 24-hour Holter monitor

Abbreviations: eGFR, estimated glomerular filtration rate; HbA1c, glycated hemoglobin; LDL; low-density lipoprotein; TG; triglyceride
